# Are We Ready to Implement Competence-Based Teaching in Pharmacy Education in Poland?

**DOI:** 10.3390/pharmacy5020025

**Published:** 2017-05-09

**Authors:** Agnieszka Skowron, Justyna Dymek, Anna Gołda, Wioletta Polak

**Affiliations:** Faculty of Pharmacy, Jagiellonian University Medical College, Krakow 30-699, Poland; jdymek@cm-uj.krakow.pl (J.D.); annagolda@cm-uj.krakow.pl (A.G.); wpolak@cm-uj.krakow.pl (W.P.)

**Keywords:** learning outcomes, pharmacy, competence framework, higher education institution

## Abstract

Pharmacists in Poland are responsible for the dispensing and quality control of pharmaceuticals. The education process in pharmacy is regulated and monitored at the national level. Pharmacy education at Jagiellonian University is organized in a traditional way based on input and content teaching. The aim of the study was to determinate whether the Jagiellonian University curriculum in the Pharmacy program meets the criteria of the European Competence Framework. The mapping of the *intended curriculum* was done by four academic teachers. The qualitative and quantitative analysis of the distribution of the European Competence Framework among a group of courses and study years was done. We observed that most of the *personal competencies* are offered to students in their senior years, while *the patient care competencies* are distributed equally during the cycle of the study, and only some of them are overrepresented at the senior years. We need a legislation change at the national level as well as organizational and mental change at the university level to move from learning outcome-based pharmacy education to competence-based.

## 1. Introduction

The Pharmacist designation in Poland is recognized in the Polish Health System as a profession responsible for the dispensing and quality control of pharmaceuticals [1]. According to the Constitution of the Republic of Poland, the pharmacist is considered as a “profession in which the public repose confidence, (…) and self-governments shall concern themselves with the proper practice of such professions in accordance with, and for the purpose of protecting, the public interest” [2]. It also constitutes pharmacists as a “regulated profession”, which is in accordance with the European Directive [3]. 

The pharmacist profession in Poland is still seen as a stable and well-paid. Analysis of the labor market showed that pharmacy graduates need only about 2–4 weeks to be employed, and during the first two years after graduation, their salaries are higher than any other medical graduates [4]. Due to the European Directive, the Master Diploma in Pharmacy (MDPharm) awarded in Poland is recognized in EU states, which improves the mobility among pharmacists and determines the competitiveness of the profession compared to other graduates [4,5]. Therefore, the main determinant which influenced the decision of young adults in choosing the pharmacy school in Poland is the confidence that in the future they will be able to find a well-paid position in Poland or in EU states [6].

Pharmaceutical education in Poland is based on the Bologna process, which regulation was implemented into the Higher Education System in Poland at the beginning of the XXI century [7,8]. As a regulated profession, the pharmacist is one of the health professions for which education is based on national standards established by law act amended by the Ministry of Science and Higher Education (MSHE) [9].

The National Standards for Pharmacy Education Act consists of five parts: (1) general requirements for pharmacy program, (2) general learning outcomes (gLO), (3) specific learning outcomes (sLO), (4) organization of the process of education, and (5) methods recommended to be used in the assessment process. The minimal requirements for the MDPharm program are the following: 11 semesters with no less than 5300 contact hours at courses and internships and 330 ECTS (European Credit Transfer System) in total. The general and specific learning outcomes are described as learning outcomes in knowledge, professional, and social skills. The specific learning outcomes are grouped into five main dimensions of sciences, such as (A) biomedical and humanistic sciences; (B) physics and chemistry; (C) analysis, synthesis, and technology; (D) biopharmacy and pharmacotherapy outcomes; (E) pharmacy practice; and (F) student’s scientific project. In Table 1, the distribution of contact hours and ECTS credits established in the national standard for pharmacy is presented in detail. The learning outcomes in the Polish National Standard for Pharmacy are described separately for knowledge and professional or social skills [9].

Despite the regulation described above, the autonomy of universities empowers academics to develop, plan, and organize the specific MDPharm program as well as to use teaching methods which ensure that student will achieve the learning outcomes established in the national standard [9].

Nowadays, among the ten Faculties of Pharmacy located in the main medical universities in Poland, approximately 1500 students graduate each year, who mainly start their professional work as pharmacists in the community and in hospital pharmacies [4,5]. In the last twenty years in pharmacy education, we observed the tendency to switch from chemistry-based pharmacy which was focused on the medicinal product, to medicine-based pharmacy which is more patient-oriented [10].

Jagiellonian University established a quality control system which aims to analyze and improve the education process to ensure that it fulfills the national standards. The Faculty of Pharmacy at Jagiellonian University Medical College (FP-JUCM) with a 250-year tradition in pharmacy teaching is one of the oldest schools of pharmacy in Central-Eastern Europe and the oldest in Poland; for the last few years, it has also been recognized as the best one in Poland [11].

The education process in the MDPharm at FP-JUCM is organized in a traditional way based on input and content teaching; this means that the student has to participate and pass the final exams of obligatory and optional courses and internships. The course syllabus contains the description of the learning outcomes and information about the teaching and evaluation methods, which are used to ensure that the student will achieve all learning outcomes. The FP-UJCM offers pharmacy students about one hundred separate courses, and half of them are obligatory. Despite obligatory courses, the student is obliged to pass at least twenty-two optional courses. Each of the obligatory courses should cover sLO described in the national standard for pharmacy. In Table 2, detailed information about the distribution of the sLO in the obligatory courses in pharmacy is shown. According to the Polish National Standard for Pharmacy, the MDPharm program covers 5.5 years of courses and internships, including six months of internship in community or hospital pharmacy [9].

A “set of competencies for pharmacists” was presented as one of the results of “Pharmacy Education in Europe—PHARMINE project” Afterwards, The PHAR-QA consortium together with the European Association of Faculties of Pharmacies extended the PHARMINE results to “produce a harmonized model for quality assurance in pharmacy education” [12]. The European Competence Framework (ECF) is one of core results of PHAR-QA project, which could be used in “setting up and/or modifying curricula in European pharmacy departments” [12]. ECF is a list of competencies for pharmacists. They consist of the two major categories—*personal competence* and *patient care competences*, which are divided into four and seven subcategories, respectively [13].

The aim of our study was to determine whether the FP-JUCM curriculum program in the MDPharm meets the criteria of the European Competence Framework [13] and to recognize the gaps and areas which need to be improved if we want our graduates to be a competent and well-educated pharmacist in the future.

## 2. Materials and Methods

The mapping process was based on “intended curriculum” of the MDPharm program designed and developed at the FP-UJCM. The MDPharm program documents consist of the *courses syllabuses* and the *program matrix table*. The *program matrix table* shows which of the obligatory courses reflect the sLO. The matrix table contains in the horizontal dimension the list of all obligatory courses and in the vertical dimension the list of sLO. The matrix is completed separately every year, and is used in quality control process to ensure that all sLO are presented in MDPharm program content.

The group of four academic teachers from FP-UJCM was involved in the mapping process. All of them were pharmacists who were awarded their Diploma in Pharmacy at Jagiellonian University. Two of the teachers were experienced academics (AS and AG) with at least ten years of experience in research and teaching in pharmaceutical sciences, and two were less-experienced (JD and WP). All teachers worked as community pharmacists in the past. Additionally, one of them (AS) was also employed in the regional office of National Fund of Health, which was a legislative and financial institution.

**Figure 1:**
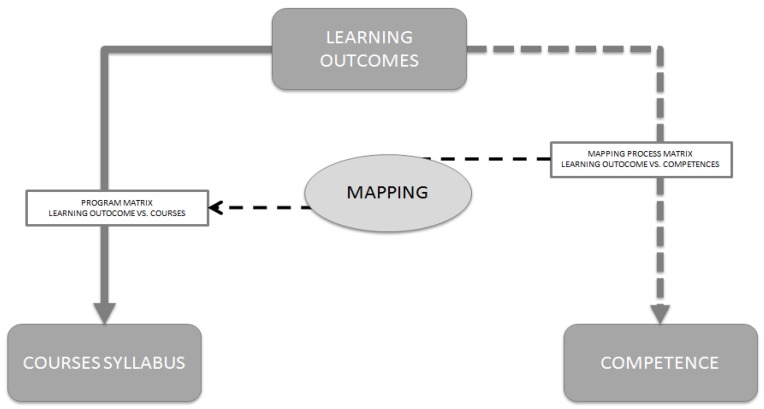
A schedule of the mapping process of the MDPharm program at FP-UJCM.

The mapping process consisted of two steps. In the first step, each academic fulfilled the matrix of competencies (in the vertical dimension) and sLO (in the horizontal dimension). So, the academics had to decide whether the sLO reflects the specific competence (from the European Competence Framework). In the second step, the matrix of competencies and sLO were translated to courses (from the MDPharm program). We use the *program matrix table* to attribute each competence to a specific obligatory course. A schedule of the mapping process is presented in Figure 1.

Finally, a quantitative analysis was done to identify gaps in the existing program. We summarize the number of courses in which learning outcomes in knowledge and skills reflect the specific competence. We also subjectively categorize the required level of the competence using the Dutch Competence Standard Framework, which consists of five levels. The gradation of students’ knowledge, skills, and professional behavior starts from level one, where the student demonstrates knowledge and basic professional behavior and ends on level five, where student “independently performs the professional activity” [14].

## 3. Results

### 3.1. Matrix of Learning Outcomes versus Competence

We assumed that a specific competence was reflected by a specific learning outcome if it was marked by at least two of the academics. The qualitative analysis of the competence vs. learning outcomes matrix showed the following:

– each competence was reflected by 23 sLO on average (the median value = 20), the maximum number of sLO reflecting the separate competence was 72, and there were two competencies which was not reflected by any of sLO; on average, competencies were reflected by 13 knowledge sLO (the median value = 10) and 10 skills sLO (the median value = 9)
– each sLO reflected three competencies on average (the median value = 2), most sLO reflected two competencies (mode)

The detailed data of some knowledge and skills sLO covering the group of competencies is presented in Table 3.

### 3.2. Matrix of Competencies versus Courses

The courses were grouped according to the scientific fields (as described in Table 3) and to the year of the study; we also included the scientific project, holiday, and final internships.

Most of the *Personal competencies in learning and knowledge* are covered by the courses in group C, which are offered mostly at final years of the study. The *Personal competencies: Values* are covered by the first and senior years of study, which offer ethics courses on the one hand, and on the other the senior internship, where the student has an opportunity to observe “real life” and to develop their attitude toward the ethical dilemma. The *Personal competencies such as communication and organization skills and research and industrial pharmacy* seem to be balanced between all groups of courses and all study years. The details of the distribution of *personal competencies* between topic groups and the years of the MDPharm are shown in Table 4.

The *Patient Care Competencies* are less covered by courses from the group B (physics and chemistry), which are mainly offered to the second year students. FP-UJCM students may achieve most of the *Patient Care Competencies* at the senior years of their MDPharm (fourth to sixth years). The details of the distribution of *Patient Care Competencies* between topic groups and years of the MDPharm are shown in Table 5. 

### 3.3. Analysis of the Level of Competencies

We based our subjective analysis (which reflects the levels of the *Dutch Competence Standard Framework*) on the document on the one hand, and our personal experience as a pharmacist and a teacher on the other. Two of our colleagues (JD and WP) could also use their experience as a pharmacy student because at least half of their courses were established basing on Bologna process. We also took into account the composition of knowledge sLO and skills sLO covering the specific competence as well as a teaching and assessing methods described in the course syllabus. The results of our discussion are presented in Table 6. In the brackets, we listed the numbers of the competencies (according to the Table 4 and Table 5) which could be achieved on the specific level at the end of the MDPharm program.

Most of the competencies (n = 12) seem to be possible to be achieved by students on the level 1 (1a to 1 c), which is a basic level and means that a student can present the knowledge and demonstrate professional behavior only in a test situation.

## 4. Discussion

The mapping process of the curriculum at the FP-UJCM was a part of the cooperation of the partners of the PHAR-QA Consortium [12], and by the discussion between partners, it was limited to “intended curriculum” mapping. We mapped the “intended curriculum” based only on official documents of the MDPharm program at our faculty, which means that the results of our analysis did not reflect the opinion of the students or another teacher. We hope it can be used to identify the gaps and to see what could be improved in future [15].

The MDPharm program at Jagiellonian University is based on learning outcomes defined at the national level [9]. It educates students to be future professional staff in a community and hospital pharmacy, so the patient-oriented European Competence Framework [13] should be widely represented and recognized in the curriculum documents.

In the first step of our analysis, we had to “translate” the sLO created for knowledge, professional, and social skills into the competencies. We observed a high inconsistency among the total number of sLO, which could be recognized as reflecting the specific *Personal competencies*. For example, we found:

– 71 sLO (56 in knowledge and 15 in skills) which we matched to competence: *Knowledge of design, synthesis, isolation, characterization and biological evaluation of active substances* (4.1 in Table 3)
– only two sLO (one in knowledge and one in skills) for competencies:
  ○ ability to identify learning needs and to learn independently (including continuous professional development (CPD)-1.1 in Table 3);
  ○ ability to critically appraise relevant knowledge and to summarize the key points (1.3 in Table 3);
  ○ ability to manage risk and quality of service issues (see 3.6 in Table 3).
– two Personal competence: *ability to apply logic to problem-solving* (1.2 in Table 3) and *ability to identify the need for new services* (3.7 in Table 3), which we could not recognize as directly represented by sLO, and consequently, delivered by any obligatory course.

A similar situation was recognized in the group of *Patient care competencies*, where:

– 72 sLO (42 in knowledge and 30 in skills) reflected the competence *Knowledge of the bio-pharmaceutical, pharmacodynamic and pharmacokinetic activity of a substance in the body* (8.1 in Table 3)
– only three sLO (in knowledge) reflected the competence *Ability to identify non-adherence to medicine therapy and make an appropriate intervention* (6.3 in Table 3) and *Knowledge of the supply chain of medicines thus ensuring timely flow of quality drug products to the patient* (8.4 in Table 3).

The analysis of the distribution of competences among the study years (Table 4 and Table 5) showed that a student has an opportunity to achieve *personal competencies* mostly during the senior years of the study (5th and 6th year). Only competencies in *research and industrial pharmacy* are distributed equally at the junior and senior years of the study. Students achieve the *patient care competencies* at the 3rd, 4th, 5th, and 6th years of the study. However, most of them—especially in the group *provision of information and services*—are distributed among the courses of the last three years (4th to 6th).

Based on the Dutch Competence Standard Framework [14], we also tried to subjectively assess the level of competencies achieved by the student [14]. In general, we assumed (Table 6) that most competencies are achieved at a level 1 or 2. There is a limited group of competencies among *Personal competencies* and *patient care competencies* which could be considered as achieved at the 4th level, and only one—*knowledge of design, synthesis, isolation, characterization and biological evaluation of active substances*—which could be achieved at a level 5. We can conclude that despite wide reflection of the *need for drug treatment* or *provision of information and service* competencies in the sLO, the subjective assessment showed that it is highly possible that a student can only present the knowledge about the specific competence and demonstrate the skills only in a test situation. This means that she is not “able to adequately carry out professional activities in an authentic professional situation under the supervision of an experienced practitioner” [14].

A major limitation of the mapping process based on “intended curriculum” is the fact that it is based on documents only, so we could not be sure that the ideas described in documents are implemented into the daily teaching activity. This means that even those competencies which we recognized as “well” reflected by the sLO might not be achieved by all students. To verify the results of our study, we plan to extend the analysis, and we are planning the study of the student's perception about their competencies.

Because the results of our analysis already showed gaps and lack of balance between competencies and learning outcomes, we will recommend Dean’s office to start the discussion with the teachers at FP-UJ CM to encourage them to switch to competence-based learning.

The main conclusion of our analysis is that the education system for pharmacy in Poland based on learning outcomes does not directly reflect the competencies. This means that to start with competence-based pharmacy education, we need to change the legal regulation at the national level and redefine our teaching at the university level. Despite the changes in the national regulations in the pharmacy field, academics should remember that their main obligation is to ensure that their graduates will be able to work independently and responsibly to improve the health of the society and to ensure the safe and effective use of drugs. As academics who are experienced in teaching, we have to be aware of our responsibility for creating the professional attitude and competencies of our students. As pharmacists and academics, we are also responsible for developing the professional education system to let our students become the professionals of the future.

## Figures and Tables

**Table 1:** The National Standard for Pharmacy—distribution of contact hours and credits in the main scientific and internship dimensions [9].

Area	Topic Group Name	Contact Hours for Student (in Total)	ECTS
Basic sciences	(A) biomedical and humanistic sciences	660	98
	(B) physics and chemistry	765	
Pharmaceutical Sciences	(C) analysis, synthesis, and technology	840	140
	(D) biopharmacy and pharmacotherapy outcomes	480	
	(E) pharmacy practice	410	
	(F) scientific project	375	
Internships	(I) holiday internships	320	10
	(IS) senior students internship (6-month)	960	40

**Table 2:** The quantitative analysis of the distribution of the specific learning outcomes (sLO) into the courses in the Master Diploma in Pharmacy (MDPharm) program at the Faculty of Pharmacy at Jagiellonian University Medical College (FP-JUCM) [9].

Courses in Specific Topic Group	Learning Outcomes (n *)		
	Knowledge	Professional Skills	Social Skills
(A) Biology/Genetics, Anatomy, Physiology, Pathophysiology, Biochemistry, Immunology, Molecular Biology, Microbiology, Botanics, First Aid, Philosophy, Psychology	32	22	3
(B) Biophysics, Inorganic and Organic Chemistry, Analytical Chemistry, Maths, Statistic, IT technology	27	17	3
(C) Medicinal Chemistry, Medicinal synthesis, Biotechnology, Pharmacognosis, Pharmaceutical Technology	41	17	-
(D) Biopharmacy, Pharmacokinetics, Pharmacology, Toxicology, Bromatology, Herbal drugs	47	69	-
(E) Pharmaceutical care, Clinical Pharmacy, Law and Ethics, Pharmacoeconomics, Epidemiology, Drug Information, Pharmacy Practice	55	55	-
(F) Scientific project	2	6	-

* number of learning outcomes in specific category.

**Table 3:** Qualitative and quantitative analysis of the distribution of the learning outcomes in the European Competence Framework (ECF) [9,12].

		Learning outcomes	
		Knowledge	Skills
1. Personal competences: learning and knowledge	1.1. Ability to identify learning needs and to learn independently (including continuous professional development (CPD)).	1	1
	1.2. Ability to apply logic to problem solving.	0	0
	1.3. Ability to critically appraise relevant knowledge and to summarise the key points.	1	1
	1.4. Ability to evaluate scientific data in line with current scientific and technological knowledge.	3	12
	1.5. Ability to apply preclinical and clinical evidence-based medical science to pharmaceutical practice.	10	14
	1.6. Ability to apply current knowledge of relevant legislation and codes of pharmacy practice.	15	9
2. Personal competences: values	2.1. A professional approach to tasks and human relations.	3	2
	2.2. Ability to maintain confidentiality.	4	2
	2.3. Ability to take full responsibility for patient care.	7	1
	2.4. Ability to inspire the confidence of others in one’s actions and advice.	8	1
	2.5. Knowledge of appropriate legislation and of ethics.	24	10
3. Personal competences: communication and organisational skills.	3.1. Ability to communicate effectively—both oral and written—in the locally relevant language.	2	4
	3.2. Ability to effectively use information technology.	3	7
	3.3. Ability to work effectively as part of a team.	5	6
	3.4. Ability to implement general legal requirements that impact upon the practice of pharmacy (e.g., health and safety legislation, employment law).	8	1
	3.5. Ability to contribute to the training of staff.	4	1
	3.6. Ability to manage risk and quality of service issues.	1	1
	3.7. Ability to identify the need for new services.	0	0
	3.8. Ability to understand a business environment and develop entrepreneurship.	2	1
4. Personal competences: research and industrial pharmacy.	4.1. Knowledge of design, synthesis, isolation, characterisation and biological evaluation of active substances.	56	15
	4.2. Knowledge of good manufacturing practice and of good laboratory practice.	29	34
	4.3. Knowledge of European directives on qualified persons.	3	3
	4.4. Knowledge of drug registration, licensing and marketing.	11	9
	4.5. Knowledge of the importance of research in pharmaceutical development and practice.	24	16
5. Patient care competences—patient consultation and assessment.	5.1. Ability to interpret basic medical laboratory tests.	6	13
	5.2. Ability to perform appropriate diagnostic tests e.g., measurement of blood pressure or blood sugar.	4	7
	5.3. Ability to recognise when referral to another member of the healthcare team is needed.	8	0
6. Patient care competences—need for drug treatment.	6.1. Ability to retrieve and interpret information on the patient’s clinical background.	25	2
	6.2. Ability to compile and interpret a comprehensive drug history for an individual patient.	5	2
	6.3. Ability to identify non-adherence to medicine therapy and make an appropriate intervention.	3	0
	6.4. Ability to advise physicians on the appropriateness of prescribed medicines and—in some cases—to prescribe medication.	17	23
7. Patient care competences–drug interactions.	7.1. Ability to identify and prioritise drug-drug interactions and advise appropriate changes to medication.	23	14
	7.2. Ability to identify and prioritise drug-patient interactions, including those that prevent or require the use of a specific drug, based on pharmaco-genetics, and advise on appropriate changes to medication.	29	13
	7.3. Ability to identify and prioritise drug-disease interactions (e.g., NSAIDs in heart failure) and advise on appropriate changes to medication.	11	14
8. Patient care competences: drug dose and formulation.	8.1. Knowledge of the bio-pharmaceutical, pharmacodynamic and pharmacokinetic activity of a substance in the body.	42	30
	8.2. Ability to recommend interchangeability of drugs based on in-depth understanding and knowledge of bioequivalence, bio-similarity and therapeutic equivalence of drugs.	24	21
	8.3. Ability to undertake a critical evaluation of a prescription ensuring that it is clinically appropriate and legally valid.	15	5
	8.4. Knowledge of the supply chain of medicines thus ensuring timely flow of quality drug products to the patient.	3	0
	8.5. Ability to manufacture medicinal products that are not commercially available.	17	7
9. Patient care competences–patient education.	9.1. Ability to promote public health in collaboration with other professionals within the healthcare system.	9	9
	9.2. Ability to provide appropriate lifestyle advice to improve patient outcomes (e.g., advice on smoking, obesity, etc.).	24	12
	9.3. Ability to use pharmaceutical knowledge and provide evidence-based advice on public health issues involving medicines.	30	8
10. Patient care competences–provision of information and service.	10.1. Ability to use effective consultations to identify the patient’s need for information.	18	9
	10.2. Ability to provide accurate and appropriate information on prescription medicines.	22	34
	10.3. Ability to provide evidence-based support for patients in selection and use of non-prescription medicines.	24	30
11. Patient care competences–monitoring of drug therapy.	11.1. Ability to identify and prioritise problems in the management of medicines in a timely and effective manner and so ensure patient safety.	12	22
	11.2. Ability to monitor and report Adverse Drug Events and Adverse Drug Reactions (ADEs and ADRs) to all concerned, in a timely manner, and in accordance with current regulatory guidelines on Good Pharmacovigilance Practices (GVPs).	21	17
	11.3. Ability to undertake a critical evaluation of prescribed medicines to confirm that current clinical guidelines are appropriately applied.	19	14
	11.4. Ability to monitor patient care outcomes to optimise treatment in collaboration with the prescriber.	18	20
	11.5. Ability to contribute to the cost effectiveness of treatment by collection and analysis of data on medicines use.	10	19

**Table 4:** Quantitative analysis of the distribution of the *Personal competencies* into the group of courses or the study year at FP-UJ CM [12].

PERSONAL COMPETENCE		GROUP A (n = 13)	GROUP B (n = 8)	GROUP A (n = 13)	GROUP D (N = 6)	GROUP E (N = 10)	YEAR 1 (N = 13)	YEAR 2 (N = 6)	YEAR 3 (N = 8)	YEAR 4 (N = 10)	YEAR 5+6 (N = 9)
		n	n	n	n	n	n	n	n	n	n
**LEARNING AND KNOWLEDGE**	1.1. Ability to identify learning needs and to learn independently (including continuous professional development (CPD)).					1					1
	1.2. Ability to apply logic to problem solving.										
	1.3. Ability to critically appraise relevant knowledge and to summarise the key points.					1				1	1
	1.4. Ability to evaluate scientific data in line with current scientific and technological knowledge.	4	1			4	4		1	2	2
	1.5. Ability to apply preclinical and clinical evidence-based medical science to pharmaceutical practice.		1		3	5	1			4	4
	1.6. Ability to apply current knowledge of relevant legislation and codes of pharmacy practice.	1				7	1			2	5
**VALUES**	2.1. A professional approach to tasks and human relations.	3				4	1	1	1		4
	2.2. Ability to maintain confidentiality.	2			1	2	1		1	1	2
	2.3. Ability to take full responsibility for patient care.	1				3			1		3
	2.4. Ability to inspire the confidence of others in one’s actions and advice.	2				5	1		1		5
	2.5. Knowledge of appropriate legislation and of ethics.	1			1	6	1			2	5
**COMMUNICATION AND ORGANISATIONAL SKILLS**	3.1. Ability to communicate effectively–both oral and written–in the locally relevant language.	1	2	1	2	2	2		2	2	2
	3.2. Ability to effectively use information technology.		4			2	3	1			2
	3.3. Ability to work effectively as part of a team.	1	3		1	3	2	1	1	2	2
	3.4. Ability to implement general legal requirements that impact upon the practice of pharmacy (e.g., health and safety legislation, employment law).					4					4
	3.5. Ability to contribute to the training of staff.	3					1		1	1	
	3.6. Ability to manage risk and quality of service issues.	1		2			1			1	1
	3.7. Ability to identify the need for new services.										
	3.8. Ability to understand a business environment and develop entrepreneurship.					1					1
**RESEARCH AND INDUSTRIAL PHARMACY**	4.1. Knowledge of design, synthesis, isolation, characterisation and biological evaluation of active substances.	2	7	5	4		5	4	2	3	3
	4.2. Knowledge of good manufacturing practice and of good laboratory practice.	8	3	7	4		5	4	5	4	3
	4.3. Knowledge of European directives on qualified persons.			4	2				2	1	2
	4.4. Knowledge of drug registration, licensing and marketing.			6	1	7			2	3	8
	4.5. Knowledge of the importance of research in pharmaceutical development and practice.	1	4	7	2	1	4	1	2	4	3

N–total number of courses in the group or study year, n–number of courses reflecting the specific competence.

**Table 5:** Quantitative analysis of the distribution of the *Patient care competencies* into the topic groups or the year of the pharmacy course at FP-UJ CM [12].

PATIENT CARE COMPETENCE		GROUP A (n = 13)	GROUP B (n = 8)	GROUP A (n = 13)	GROUP D (N = 6)	GROUP E (N = 10)	YEAR 1 (N = 13)	YEAR 2 (N = 6)	YEAR 3 (N = 8)	YEAR 4 (N = 10)	YEAR 5+6 (N = 9)
		n	n	n	n	n	n	n	n	n	n
**PATIENT CONSULTATION AND ASSESSMENT**	5.1. Ability to interpret basic medical laboratory tests.	5	1	1		1	2	2	3		1
	5.2. Ability to perform appropriate diagnostic tests e.g., measurement of blood pressure or blood sugar.	4	1	1			2	2	2		
	5.3. Ability to recognize when referral to another member of the healthcare team is needed.	1			2	4			1	2	4
**NEED FOR DRUG TREATMENT**	6.1. Ability to retrieve and interpret information on the patient’s clinical background.	9		2		2	3	3	3	1	2
	6.2. Ability to compile and interpret a comprehensive drug history for an individual patient.	1		1	1	2			1	2	2
	6.3. Ability to identify non-adherence to medicine therapy and make an appropriate intervention.					2					2
	6.4. Ability to advise physicians on the appropriateness of prescribed medicines and–in some cases–to prescribe medication.	1	1	5	5	6	1		3	5	8
**DRUG INTERACTIONS**	7.1. Ability to identify and prioritise drug-drug interactions and advise appropriate changes to medication.	7		1	5	2	3	2	4	3	3
	7.2. Ability to identify and prioritise drug-patient interactions, including those that prevent or require the use of a specific drug, based on pharmaco-genetics, and advise on appropriate changes to medication.	6		3	5	2	2	2	4	3	4
	7.3. Ability to identify and prioritise drug-disease interactions (e.g., NSAIDs in heart failure) and advise on appropriate changes to medication.			3	5	2			2	3	4
**DRUG DOSE AND FORMULATION**	8.1. Knowledge of the bio-pharmaceutical, pharmacodynamic and pharmacokinetic activity of a substance in the body.	9	1	6	5	2	4	3	6	5	4
	8.2. Ability to recommend interchangeability of drugs based on in-depth understanding and knowledge of bioequivalence, bio-similarity and therapeutic equivalence of drugs.			6	6				3	5	3
	8.3. Ability to undertake a critical evaluation of a prescription ensuring that it is clinically appropriate and legally valid.			5	4	2			3	3	4
	8.4. Knowledge of the supply chain of medicines thus ensuring timely flow of quality drug products to the patient.					2					2
	8.5. Ability to manufacture medicinal products that are not commercially available.	1		5	3	1	1		3	2	3
**PATIENT EDUCATION**	9.1. Ability to promote public health in collaboration with other professionals within the healthcare system.	4			2	2		1	3	1	3
	9.2. Ability to provide appropriate lifestyle advice to improve patient outcomes (e.g., advice on smoking, obesity, etc.).	9			2	1	3	3	3	2	1
	9.3. Ability to use pharmaceutical knowledge and provide evidence-based advice on public health issues involving medicines.	6			3	4	2	2	2	4	3
**PROVISION OF INFORMATION AND SERVICE**	10.1. Ability to use effective consultations to identify the patient’s need for information.	5			2	5	3		2	3	4
	10.2. Ability to provide accurate and appropriate information on prescription medicines.	2		5	5	5	1		3	5	7
	10.3. Ability to provide evidence-based support for patients in selection and use of non-prescription medicines.	2		5	5	5	1		3	4	8
**MONITORING OF DRUG THERAPY**	11.1. Ability to identify and prioritise problems in the management of medicines in a timely and effective manner and so ensure patient safety.	1		3	4	4	1		2	3	6
	11.2. Ability to monitor and report Adverse Drug Events and Adverse Drug Reactions (ADEs and ADRs) to all concerned, in a timely manner, and in accordance with current regulatory guidelines on Good Pharmacovigilance Practices (GVPs).			4	4	4			1	6	5
	11.3. Ability to undertake a critical evaluation of prescribed medicines to confirm that current clinical guidelines are appropriately applied.		2	5	6	7	2		3	7	8
	11.4. Ability to monitor patient care outcomes to optimize treatment in collaboration with the prescriber.	1	1	5	5	6	2		2	6	8
	11.5. Ability to contribute to the cost effectiveness of treatment by collection and analysis of data on medicines use.		4	4	2	4	3	1	2	4	4

N–total number of courses in the group or study year, n–number of courses reflecting the specific competence.

**Table 6:** A desk analysis of the level of competence achieved by a student at the MDPharm program at FP-UJCM [14].

		Level *
**Personal Competencies**	learning and knowledge	1a (1,3,4); 1c (5,6)
	values	2 (3,4); 3 (5); 4 (1,2)
	communication and organisational skills	1a (1,3); 1c (4); 2 (6); 3 (7,8); 4 (2)
	research and industrial pharmacy	1c (3,5); 3 (2,4); 5 (1)
**Patient Care Competencies**	patient consultation and assessment	1a (1,3)
	need for drug treatment	1a (2,3); 1c (1,4)
	drug interactions	1a (1); 2 (2,3)
	drug dose and formulation	1c (2); 2 (5); 3 (1,4); 4 (3)
	patient education	1c (2); 2 (3); 3 (1)
	provision of information and service	1c (1); 2 (2,3)
	monitoring of drug therapy	1a (3,5); 2 (1); 4 (2,4)

* the level of Dutch Competence Standard Framework; in the brackets, we used the numbers of the specific competencies from Table 4 and Table 5.
